# Emoji Identification and Emoji Effects on Sentence Emotionality in ASD-Diagnosed Adults and Neurotypical Controls

**DOI:** 10.1007/s10803-022-05557-4

**Published:** 2022-04-12

**Authors:** Christopher J. Hand, Ashley Kennedy, Ruth Filik, Melanie Pitchford, Christopher M. Robus

**Affiliations:** 1 School of Education, University of Glasgow, 11 Eldon Street, Glasgow, G3 6NH UK; 2 Department of Psychology, Glasgow Caledonian University, Glasgow, UK; 3 School of Psychology, The University of Nottingham, Nottingham, UK; 4 School of Psychology, University of Bedfordshire, Luton, UK; 5 School of Psychotherapy and Psychology, Regents University London, London, UK

**Keywords:** Autism spectrum disorders, Double empathy, Emoji, Emotion, Social information processing

## Abstract

We investigated ASD-diagnosed adults’ and neurotypical (NT) controls’ processing of emoji and emoji influence on the emotionality of otherwise-neutral sentences. Study 1 participants categorised emoji representing the six basic emotions using a fixed-set of emotional adjectives. Results showed that ASD-diagnosed participants’ classifications of fearful, sad, and surprised emoji were more diverse and less ‘typical’ than NT controls’ responses. Study 2 participants read emotionally-neutral sentences; half paired with sentence-final happy emoji, half with sad emoji. Participants rated sentence + emoji stimuli for emotional valence. ASD-diagnosed and NT participants rated sentences + happy emoji as equally-positive, however, ASD-diagnosed participants rated sentences + sad emoji as more-negative than NT participants. We must acknowledge differential perceptions and effects of emoji, and emoji-text inter-relationships, when working with neurodiverse stakeholders.

**Supplementary Information:**

The online version contains supplementary material available at 10.1007/s10803-022-05557-4.

## Introduction

Accurate facial-emotion recognition is fundamental in many contexts, and especially within reciprocal relationships. Accompanied by physiological responses, emotions are purposefully or passively conveyed through conduct, behaviour, and expression (Leman et al., 2012). For neurotypically (NT) developed individuals, human faces provide unparalleled sources of socio-emotional data (Farah et al., 1998; Kanwisher et al., 1996). However, for individuals with a diagnosis of Autism Spectrum Disorder (ASD), differences in the fusiform face area (FFA) and amygdala influence gaze and memory, influencing the interpretation and response to non-verbal cues of others (e.g., Baron-Cohen et al., 2000; Golarai et al., 2006; Pelphrey & Carter, 2008; Schultz, 2005). Research suggests that emotion recognition differences are common within populations of individuals with an ASD diagnosis (Hobson, 1986). Challenges in distinguishing emotions can be affiliated with reductions in life-satisfaction and interpersonal difficulties (Carton et al., 1999). The purpose of the current research was to explore how ASD-diagnosed adults and NT controls classify emoji representing the six basic emotions (Study 1), and whether the effects of emoji on the perceived emotionality of short narrative texts differ across participant groups and across the emoji used (Study 2).

### Emotion Recognition Differences in ASD

Emotion recognition typically relies on holistic processing, utilizing spatial configuration of primary features (Diamond & Carey, 1986; Farrah et al., 1998; Valentine, 1988). Fridlund’s (1994) behavioural ecological perspective postulates that the human ability to interpret and express facial emotional cues serves as both an inference and predictor of behavioural intent (Adams et al., 2006). Such cognitive and emotional proficiencies provide most humans with the ability to empathise (Baron-Cohen et al., 2003). Ekman and Friesen (1971) maintained that emotion directly reflects emotions *felt*, as opposed to conveyance of behavioural intent (Fridlund, 1994). Following Ekman and Friesen (1971), researchers delineated six basic cross-culturally identifiable expressions: happiness, disgust, fear, sadness, surprise, and anger. The communicative function of emotions has been established as integral within dynamic reciprocal relationships, and impairment of this ability—as proposed in ASD (Thye et al., 2018)—has been scrutinised.

Described as a pervasive neuro-developmental condition, ASD affects 1 in 160 children worldwide (World Health Organisation, 2004; WHO). A genetically-inclined, neuro-developmental paradigm has been proposed to underpin autism (Folstein & Rosen-Sheidley, 2001), supported by the identification of unique patterns of brain development and activity in individuals with a diagnosis of ASD (e.g., Hill et al., 2004). Many such individuals are prone to systemising—the rule-governed inductive process of data-gathering, quantifying differences and correlations to generate predictable results (Kidron et al., 2018). Systemising is typically associated with males, object processing, and is applicable to phenomena which are lawful, finite, and deterministic (Baron-Cohen et al., 2003). Lawson et al. (2004) demonstrated that ASD was associated with systemising and divergence from empathising.

However, some of the ‘stereotypical’ findings in this area are problematic. A recent editorial by Fletcher-Watson and Bird (2020) deconstructs relationship between autism and empathy. From the offset, the authors clearly identify that a major obstacle is that there is no single standardised unequivocal researcher definition of empathy/empathising. Young autistic children with concomitant intellectual disability have been found to be more likely to fail to detect another person’s emotional cues, due to differential orienting strategies in these children (Fletcher-Watson & Bird, 2020; Mundy, 2018). Chita-Tegmark (2016) suggests that such differences might extend to adults, although this has been challenged (e.g., Johnson, 2014). Individuals not only have to perceive the emotional expressions/behaviours of another, they must be able to correctly identify this information correctly, and Harms et al. (2010) have suggested that this is more difficult for autistic people. If emoji are used in interpersonal communication by senders to communicate their own emotional states, are these emoji: ‘recognised’ similarly by autistic and non-autistic individuals?, and are the effects of emoji on the perceived sentiment of written texts the same for autistic and non-autistic people?

Another part of the process, as described by Fletcher-Watson and Bird (2020) is the embodiment of the emotional signals of another person—that is, experiencing the same emotions. Finally, an autistic person might be perceived as non-empathetic due to their responses to the emotional situation they are involved in. Fletcher-Watson and Bird (2020) argue that this is not the outcome of a ‘lack of empathy’; rather, the autistic person is merely following a different “response-script” to that of an NT individual (Fletcher-Watson & Bird, 2020, p. 3). Milton (2012) has suggested a *double empathy problem* underlying patterns of research and real-world data. That is, challenges around communication and understanding between autistic and non-autistic people should not be seen as one-sided—rather, these complications are resultant from different perspectives of the communicators. For example, Edey et al. (2016) and Sheppard et al. (2016) demonstrate the non-autistic participants demonstrated difficulties when attempting to evaluate the emotional expressions of autistic persons (Fletcher-Watson & Bird, 2020).

Currently, a fully neurobiological model of ASD is lacking (Sivaratnam et al., 2015); hence, ASD is predominantly explained via cognitive models. Historically, conversations around differences in Theory of Mind (ToM; Premack & Woodruff, 1978) abilities were predominant. ToM outlines one's capacity to predict mental states, and thus actions, intentions, and beliefs of those around them (Frith & Frith, 2003; Wang, 2015; Wellman, 1992). Difference in ToM abilities between ASD and NT samples have been suggested by studies of cognition-based emotion recognition (Baron-Cohen et al., 1993). However, such claims have been contested, suggesting that results may have arisen from experimenter bias and social conditioning (Fiene & Brownlow, 2015; Said et al., 2011). Chevallier et al. (2012) argued that children diagnosed with ASD perform poorly in ToM tasks administered by a researcher in a face-to-face context; such testing constructs a social situation, thus misrepresenting the performances of participants with ASD relative to NT controls. Chevallier et al. examined this by administering the false-belief test via computer, as opposed to in-person. Although NT individuals outperformed participants with ASD in traditional researcher-administered trials, no difference was found between-groups when administered via computer. This implies sensitivity differences to social situations only.

Indeed, researchers have recently begun to partial out the variability associated with alexithymia and autism. Alexithymia and autism are distinct but potentially co-morbid considerations (Fletcher-Watson & Bird, 2020). Alexithymia is characterised by difficulties in identifying emotional arousal and feelings (Nemiah et al., 1976). Alexithymia affects approximately 50% of individuals with autism (Bird & Cook, 2013), as opposed to 10% of the general population (Salminen et al., 1999). Previous research posits that alexithymia might underlie the stereotypical impairment of emotion recognition in ASD populations (e.g., Cook et al., 2013; Grynberg et al., 2012; Ketelaars et al., 2016; Swart et al., 2009) and has led to the formulation of the Alexithymia Hypothesis (Bird & Cook, 2013). Work by Brewer et al. (2015) suggests that autism may be associated with non-typical ToM but not impaired empathy, whereas alexithymia may be associated with non-typical empathy but not atypical ToM (Brewer et al., 2015; Fletcher-Watson & Bird, 2020).

Autism has been framed by an interest model—that is, characterised by monotropic attention strategies, repetitive behaviours and interests, and attentional ‘tunnelling’ (e.g., Lawson, 2010; Murray et al., 2005). Monotropic theories posit that autism is defined by ‘single-minded’ attentional systems, with selects one information source at a time, which might result in certain social cues being neglected if another source of information is more-salient (Fletcher-Watson & Bird, 2020; Murray et al., 2005).

Historically, it was believed that emotion recognition was unequivocally impaired in individuals with ASD, through failure to accurately comprehend others’ emotional states (Hobson, 1986). This has been countered by data from studies with well-matched pairs (e.g., Ozonoff et al., 1990). Pelphrey et al. (2002) utilised photographs representing the six basic emotions. Their study consisted of two phases: in the first, visual scan paths were examined whilst ASD and NT participants viewed images; in the second, emotion identification accuracy between-groups was compared. Five male, high-functioning ASD-diagnosed and five male NT participants were recruited. ASD-diagnosed participant scan-path analyses were consistent with highly variable viewing patterns of external facial features (e.g., ears, chin, hairline); NT controls showed consistent strategic paths over internal facial features (e.g., eyes, nose, mouth). Phase two demonstrated differences in emotion-recognition accuracy between-groups, with greater judgement-diversity evident in ASD-diagnosed participants. Although seemingly confirmatory of NT individuals outperforming their ASD-diagnosed peers, only fear recognition was significantly different—most-commonly mistaken for disgust or anger*.* Significant differences were not observed for the remaining five emotions. These results were obtained from small samples, with a lack of matched-pairs, and all-male participant pool.

Uljarevic and Hamilton’s (2012) meta-analysis encompassed 48 studies (*N* = 980) involving ASD-diagnosed participants. Of these, 28 utilised Ekman and Friesen’s (1976) facial affect stimuli. Studies incorporating measures of full-scale intelligence quotients (FSIQs) and a wide participant age range (6–41 years) were analysed. This meta-analysis found no significant difference among ASD-diagnosed participants in happiness recognition (applied as a baseline in the absence of neutral face data), sadness, surprise, disgust, or anger. Fear was acknowledged as less-accurately recognised than happiness, but with only marginal significance. Uljarevic and Hamilton’s (2012) evaluation demonstrated surprise was no more misperceived than any other emotion; however, they acknowledge complexities in drawing comparisons between the emotions most- and least-accurately recognised, given that only eight studies compared all six emotions.

Uljarevic and Hamilton (2012)’s meta-analysis found no effects of age or IQ on emotion recognition; hence, recognition differences are not necessarily subgroup-specific for ASD-diagnosed individuals (e.g., “lower-functioning” individuals). Studies which matched participants on IQ were, at best, indicative of ASD-diagnosed participants preforming at the expected level for their *mental* age, as opposed to analogous with individuals of the same *chronological* age. Consistent performance in happiness recognition appears to oppose a universality of atypicality; poorer fear recognition aligns with theories associating reduced eye-contact and poorer amygdalaic fear processing. Uljarevic and Hamilton (2012) propose that previous findings may be mediated by stimulus timings—given ASD-diagnosed individuals’ divergent looking-patterns, results may reflect limited processing-time rather than recognition difficulties. Collectively, results indicate atypical facial processing in ASD-diagnosed samples, suggesting a mechanism which actualises social information processing differences in ASD.

### Emotion Recognition and Online Communication

Mazurek (2013) argues that a reduction in peer-engagement for ASD-diagnosed individuals is associated with decreased life-satisfaction, increased anxiety, depression, and low self-esteem. Social media provides opportunities for ASD-diagnosed individuals to interact with peers in environments void of non-verbal communicative cues, having less socially-regimented rules of engagement, lack of eye-contact, and reduced reliance on non-verbal cues of facial affect and emotional decoding (Burke et al., 2010). Emoji are frequently used in online interactions and communications, as a proxy for face-to-face interactions.

Emoji are pictorial images which can mimic facial expressions and are considered a paralinguistic medium through which attitudes, emotions and narratives are shared, often in conjunction with written text (Rodrigues et al., 2017). Kaye et al. (2017) state that emoji serve two primary functions: (i) portray emotional or social intent, (ii) reduce potential discourse ambiguity. Social Information Processing theory (Walther, 1992) states that *cues* within online communications (amongst which emoji can be considered) develop and maintain relationships (Rodrigues et al., 2017). Skovholt et al. (2014) highlight that emoji function as context cues, attitude markers and social relationship organisers (e.g., decreasing formality). Research has shown both face- and face-emoji-related activation of the occipital-temporal cortex (Churches et al., 2014), suggesting that via associative learning, emoji processing lies parallel to human facial processing, and the associated emotion represented (Bai et al., 2019).

In contrast to human face-processing research, studies suggest that ASD-diagnosed individuals are adept at recognising cartoon faces (Rosset et al., 2007; van der Geest et al., 2001). Atherton and Cross (2018) highlight that ASD-diagnosed participants showed increased engagement with anthropomorphic images. Attentional bias research has shown that ASD-diagnosed individuals demonstrate increased fixation on cartoon-style characters, relative to real objects (van der Geest et al., 2001). Hence, it may be presumed the use of cartoon-type faces (i.e., emoji) influence emotion recognition abilities in ASD-diagnosed populations.

### Emoji and Language Processing

Emoji are frequently used alongside written language. Similar to emotion recognition, ToM can be aligned with text valence processing, stating that text comprehension depends on readers’ capacity to attribute others’ cognitive and affective states (Abu-Akel & Shamay-Tsoory, 2011). Emoji might be of benefit when considering Milton’s (2012) double empathy problem—that is, the emoji might simultaneously enable the sender to convey their emotional state/intention and serve as a cue to the recipient to aid their own perception of the message and select an appropriate response more clearly. Pictorial representations may aid text interpretation (Walther & D’Addario, 2001); Derks et al. (2007) and Lo (2008) demonstrated that emoticons strengthened emotional sentiment of texts, biasing readers toward emoticon valence. González-Ibáñez et al. (2011) and Muresan et al. (2016) found that emoticons were influential in classifying sarcastic, non-sarcastic, positive, and negative tweets. Thompson and Filik (2016) stated that emoticons can reduce negative responses typically experienced in response to ironic texts. Walther and D’Addario (2001) used artificially-created emotive emails containing either positive or negative emoticons. However, results indicated valence perceptions were unaffected, implying emoticons’ emotional influence was overshadowed by text sentiment—except for negative text accompanied by negative emoticons.

Many studies have focused primarily on conversational formats involving response dialogues (Riordan & Kreuz, 2010; Rodrigues et al., 2017). Questions remain as to whether emoji impact on different texts, i.e., narrative sentences composed from an external third-person perspective. Willoughby and Liu (2018) compared narrative and non-narrative sentences via iMessage conversations, containing either three (high frequency), one (low frequency), or no emoji. Results suggest that iMessages without emoji elicited greater levels of credibility and elaboration, whereas a higher number of emoji drew greater attentional focus, regardless of narrative format presented.

Robus et al. (2020) examined the effects of emoji position and expression in neutral narrative sentences on eye movements during reading and subjective ratings of sentence emotional valence. Pre-tested neutral sentence stimuli were used, allowing for a purer measurement of emoji effects. Two emoji were used, identical in colour and formatting, which differed in expression—slightly smiling () and slightly frowning (). Emoji influence on text valence was predominantly non-significant; this may have been a result of the lack of ‘strength’ of the emoji used and the artificial laboratory eye-tracking set-up.

### The Current Research

The world around is becoming increasingly digitised, and this process is if anything accelerating. Emoji are becoming ubiquitous in interpersonal electronic communication—digital interpersonal communication is more likely to involve an exchange of text + emoji as opposed to text + emoticon (e.g., Boutet et al., 2021; Sampietro, 2020). Interpersonal communication relies upon verbal and non-verbal information, and there is extant evidence to suggest that non-verbal information shapes social perceptions (e.g., Frith & Frith, 1999; Willis & Todorov, 2006). Thus, it is important that we understand how emoji are processed by different stakeholders, and what the potential emoji type × user ‘typicality’ interaction is on understanding and perceptions. We present two studies. In Study 1, we examined ASD-diagnosed and NT participants’ identifications of the six basic emotions, as depicted by emoji. In Study 2, we considered the effect of emoji on perceptions of otherwise neutral narrative texts, and the possibility of differential effects of happy/sad emoji on ASD-diagnosed and NT participants’ text-sentiment perceptions.

## Study 1

Study 1 examined whether emotion recognition differences in ASD-diagnosed individuals extend to the online environment. Identification accuracy for Ekman et al.’s (1972) six basic emotions, conveyed via emoji, was compared between ASD-diagnosed individuals and NT controls. Research suggests encoding of emoji have been shown to be representative of encoding of facial emotion (Churches et al., 2014; Rosset et al., 2007; van der Geest et al., 2002). Thus, it was predicted that ASD-diagnosed participants would present significantly lower accuracy scores when identifying emotion through emoji in comparison to NT individuals.

## Method

### Participants

An a priori power analysis calculated using G*Power 3.1, with an anticipated effect size of *f* = 0.25, an α = 0.05, and desired power of 0.90 (Cohen, 1988) suggested a total target sample size of 72 participants. Recruitment consisted of both online advertising and posters circulated throughout [HOST UNIVERSITY] campus. Eighty-eight adults took part; 18 identified as male (*M*_age_ = 25.61 years; SD_age_ = 5.42), 68 as female (*M*_age_ = 28.88 years; *SD* = 11.45), and two did not disclose their gender-sex (*M*_age_ = 35.00 years; SD_age_ = 9.90 years). In this study, 31 participants confirmed as having formally received an ASD diagnosis (10 males, *M*_age_ = 24.90, SD_age_ = 6.06; 19 females, *M*_age_ = 31.42, SD_age_ = 12.81; 2 non-disclosed, *M*_age_ = 35.00, SD_age_ = 9.90). Recruitment materials and the participant information sheets were designed to explain in plain language what was required to be considered as ‘diagnosed’. We stated that diagnosis must have been provided in writing by a professional/on behalf of a team of professionals, and that this process should have involved healthcare professionals, and/or educators, and/or psychologists, and/or professionals allied to autism support agencies. Ethical and practical considerations prevented further verification of diagnosis (e.g., disclosure of medical records, specific details of the person or persons involved in the diagnostic process). A further 57 participants were otherwise NT (8 males, *M*_age_ = 26.50, SD_age_ = 4.75; 49 females, *M*_age_ = 27.88, SD_age_ = 10.86).

### Design, Materials, and Procedure

A 2 (group: *ASD, NT*) × 6 (emoji type: *happy, disgusted, fearful, sad, surprised, angry*) mixed-factors design was implemented via an online survey method to investigate participants’ accuracy in identifying emotions expressed by emoji. Emoji were selected from the Common Locale Data Repository (CLDR version 13, Kaye et al., 2017; http://cldr.unicode.org/). Twelve stimuli were selected: two emoji depicting each of Ekman et al.’s (1972) six basic emotions. To mitigate between-user familiarity, one of each emoji type was iOS format and one was Android format; all emoji were identical in size and highly comparable in colour (Fullwood et al., 2013; Oleszkiewicz et al., 2017; Wolf, 2000). In the absence of an emoji specifically labelled with the target emotion, emoji were selected via matching of prominent key features, for example, *disgusted*—characterised by a drawn-in mouth and lowered eyebrows. Although this is a relatively small item set, the linear mixed effect modelling process used to analyse the data acknowledges each single observation individually, rather than collapsing these onto by-subject or by-item amalgamations as an analysis of variance (ANOVA) would do. Thus, we retain a larger amount of power even with a moderate item set. Furthermore, given that our autism-diagnosed participants would potentially be frustrated by this task and might have found it especially difficult, it was considered an ethical risk to deploy a substantially larger item set (a practical consideration regarding the attrition risk for *every* participant also fed into this decision). See Table 1 for indicative stimuli.

**Table 1:**
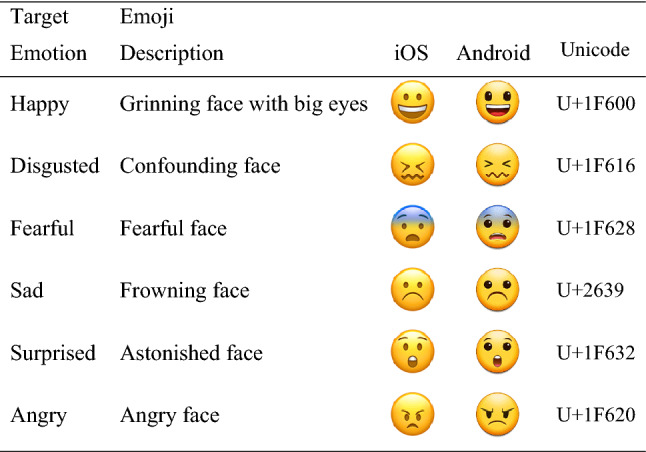
Target emoji stimuli

The study was hosted by SurveyHero (https://www.surveyhero.com/). British Psychological Society guidelines were adhered to, regarding ethical research and conduct (BPS, 2014). Prior to completing the emoji identification task, participants were informed as to the purpose of the study, before providing consent. Demographic information was collected, including age, gender-sex, and participant group identification, before instructions on how to complete the emoji identification task. Participants were presented with each emoji—one-at-a-time—and were asked to choose one option from a list of six emotional adjectives (*happy, disgusted, fearful, sad, surprised, angry*), indicating which emotion they thought was being expressed/represented. Stimuli were presented in a random order for each participant. Each stimulus was displayed for an unlimited amount of time (i.e., until participants executed their response). Participants had no time-restriction for viewing/rating stimuli as Uljarevic and Hamilton (2012) indicate that time-restrictions negatively influence task-performance within ASD-diagnosed samples. Participants did not receive feedback as to their response accuracy. Upon completion, participants were debriefed and provided with contact details of the lead researcher, supervisor, and relevant external organisations (e.g., UK National Autistic Society; https://www.autism.org.uk/).

## Results

Participant accuracy was determined on a trial-by-trial basis, with either a 1 (correct identification) or a 0 (incorrect identification) coded. Across participants and trials, there were 1,056 data points available for analysis. We used the ‘lme4’ R package (Bates et al., 2015; R Development Core Team, 2016; http://www.r-project.org); we followed a generalized linear mixed-effects approach using the ‘glmer’ command and added the argument “*family* = *binomial*”, given the nature of our accuracy data. Optimal random effect structures were identified using forward model selection (see Barr et al., 2013; Matuschek et al., 2017). Fixed effects were tested using likelihood-ratio tests comparing full and reduced models. Post-hoc tests were conducted using the ‘emmeans’ package (v1.4.8, 26/06/20; Lenth et al., 2020), and significance thresholds adjusted using the Tukey method. Descriptive statistics are presented in Table 2.

**Table 2:**
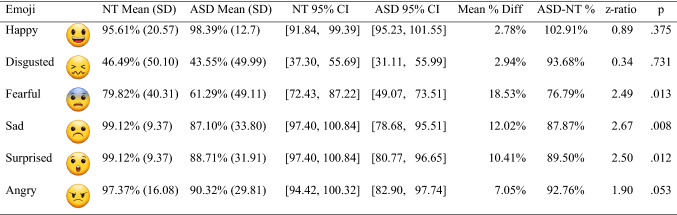
Mean (standard deviation) accuracies and 95% confidence intervals across groups and emoji types3

Figures (except *p*-values) rounded to 2 decimal points. ASD-NT % = relative performance of ASD group to NT group, calculated as (ASD mean/NT mean) × 100

Our model included random intercepts by participants [*χ*^2^ = 24.00, *df* = 1, *p* < 0.001] and by trials [*χ*^2^ = 231.11, *df* = 1, *p* < 0.001]. There was a significant effect of participant group on emoji identification accuracy [*χ*^2^ = 8.13, *df* = 1, *p* = 0.004]; overall, NT participant accuracy was 86.26%, whereas ASD-diagnosed participant accuracy was 78.23%. A significant effect of emoji type was also found [*χ*^2^ = 31.33, *df* = 5, *p* < 0.001]; planned follow-up comparisons revealed that *disgusted* emoji identification was significantly poorer than all other emoji [all *p*s < 0.001], and that *fearful* emoji identification was poorer than *happy, sad, surprised*, and *angry* [all *p*s < 0.003]. No other comparisons were significant [all *p*s > 0.307].

**Fig. 1:**
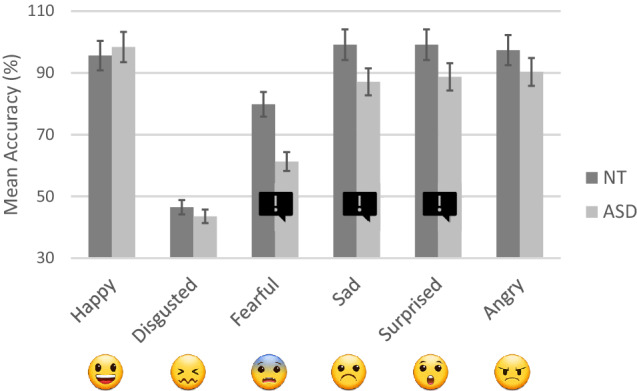
Mean accuracies (5% error bars) across emoji and groups.  indicates a significant difference between groups

A significant group × emoji type interaction was observed [*χ*^2^ = 19.21, *df* = 5, *p* = 0.002]. Between-group comparisons across emoji types are summarised in Table 2 and illustrated in Fig. 1.

Planned follow-up comparisons revealed that NT and ASD-diagnosed participants were equally consistent when identifying *happy, disgusted,* and *angry* emoji. NT participants were significantly more typical than ASD-diagnosed participants when identifying *sad, fearful*, and *surprised* emoji [*p* = 0.0127, 0.0075, and 0.0123, respectively].

We compared the effect of emoji type on identification accuracy within each group. These comparisons are presented in Supplementary Material A, Table A1. The key finding was that NT participants were better-able to distinguish *fearful* emoji from *disgusted* emoji, however there was no such difference in ASD-diagnosed participants’ responses.

We considered the breakdown of participants’ incorrect response-choices, across groups and emoji types. A full breakdown of this data (a confusion matrix) is presented in Supplementary Material B, Table B1. Points of note were that when NT participants misperceived disgust, the most-common selection was anger, whereas ASD-diagnosed participants incorrectly chose fear. Misperceptions of fear-as-surprise represented most errors in the classification of the fearful emoji, but in a far greater proportion of ASD-diagnosed participants than NT controls. Among ASD-diagnosed participants, sadness was most-commonly mistaken for fear (closely followed by disgust), and a similar pattern was seen in ASD-diagnosed participants error data for the surprise emoji. Finally, the error data for the angry emoji suggested that ASD-diagnosed participants were more likely to mis-associate this emoji with disgust than any other alternative. No other emoji was mis-perceived as ‘happiness’, in either participant group.

## Discussion

We predicted a between-group difference among ASD-diagnosed and NT participants when identifying the six basic emotions in emoji form. Analyses demonstrated between-groups differences in three of the six emotions (fear, sadness, surprise). Non-significant group differences were observed for happiness, disgust, and anger. Previous research had only identified significant group differences in the recognition of visualisations of fear (Pelphrey et al., 2002; Uljarevic & Hamilton, 2012). Our ASD participants were highly accurate when recognising happiness. The greatest difference between-groups was observed for fear recognition. Overall, the results of Study 1 are consistent with emotion perception/categorisation differences and diversity within ASD-diagnosed individuals.

### Implications and Influencing Variables

Our results suggest that certain differences between autistic and non-autistic individual in relation to the processing of emotional information and emotional cues might extend to emoji (e.g., Chita-Tegmark, 2016; Harms et al., 2010). The precise mechanisms underlying these patterns of effects are difficult to ascertain. It may be posited that face and emoji processing yield comparative effects, representing similar processing; both tasks activate the occipital-temporal cortex (Churches et al., 2014). Our results oppose the argument that anthropomorphic representations of emotions are more-efficiently recognised than human faces in ASD populations (Rosset et al., 2007). Reduced accuracy related to emotion comprehension, with moderate-to-large effect sizes observed, implying that similar results would be observed from different, comparable samples.

It may be that results were an artefact of providing multiple choice responses to participants. Labelling tasks require verbal skills and forced-choice labelling may enable participants to guess correct answers (Frank & Stennett, 2001). This was accounted for in Uljarevic and Hamilton’s (2012) meta-analysis, finding forced-choice labelling to have no overall influence on performance, re-enforcing that ASD-diagnosed participants’ accuracy impairments were due to emotional processing, rather than linguistic task-demands. Furthermore, we limited our stimulus set to 12 items for practical and ethical reasons. Although the appropriate sample size and powerful cutting-edge linear mixed effects analysis mitigates this, we would of course hope to continue this research with a more expansive stimulus set. By including more trials with (multiple) different emoji that represent the six basic emotional expressions, we can understand more about the similarities and differences between neurodivergent and NT individuals.

The results of Study 1 show a difference in emoji perceptions of individuals with an ASD diagnosis and NT individuals. In everyday life, emoji are used in conjunction with written language (at the end of text messages, in email, etc.). With the increased digitisation of all facets of society, it is important to understand how emoji influence the perception of any accompanying messaging. Having established in Study 1 that ASD-diagnosed and NT groups process emoji differently, we considered how emoji influence the perceived emotionality of short narrative texts for both ASD-diagnosed and NT participants.

## Study 2

Emoji can enrich verbal expression and can enable greater emotional expressiveness in the absence of social contextual markers. There is an insufficient evidence-base regarding how emoji influence written language processing, and what work has been done is often limited by non-optimal analytical methods (e.g., Boutet et al., 2021). Study 2 revisits the work of Robus et al. (2020), which typically found non-significant effects of emoji on perceived narrative text valence. Robus et al. generated a controlled set of narrative text stimuli, pre-testing for emotional-neutrality prior to adding emoji. In Study 2, we use these same neutral narrative sentences. Robus et al. acknowledged that their positive emoji () may not have been ‘positive enough’ to influence valence perception, and that only emoji from one platform were used. We used the same happy/sad emoji as in Study 1 (see Table 1), representing two different platform styles (iOS, Android).

Most prior work in this area has used general linear model-type analyses, despite outcomes being measured ordinally, and/or the assumptions of the analyses being violated. Likert scales are ordinal by nature; although scale points might appear equally spaced and equivalent, there is no evidence that every participant agrees as to what constitutes a response at each scale point, or that each participant’s evaluations of adjacent points are equal (Taylor et al., 2021). The relationship between participant responses and underlying latent dimension(s) are at-best underspecified (Taylor et al., 2021). The analytical approach used in Study 2—cumulative link mixed modelling (CLMM)—maps ordinal outcomes against ordered regions of a latent distribution (Bürkner & Vuorre, 2019; McCullagh, 1980). We utilised this approach to estimate the fixed effects (participant group, emoji type, group × emoji type), embracing the ‘randomness’ generated by individual participants and items included in the study (Taylor et al., 2021). There is clear evidence of the problems in using general linear modelling/ANOVA to evaluate ordinal data sets, and the need for CLMM approaches within experimental studies has been successfully argued by, for example, Liddell and Kruschke (2018).

We predicted that emoji would bias perceived emotionality in the ‘direction’ of the emoji (happy-positive, sad-negative). We considered between-group differences between ASD-diagnosed and NT participants. We predicted a group × emoji type interaction on sentence ratings—based on the results of Study 1, we did not expect a between-group difference for happy emoji sentences ratings; however, we anticipated a between-group difference for sad emoji sentence ratings.

## Method

### Participants

An a priori power analysis calculated using G*Power 3.1, with an anticipated effect size of *f* = 0.25, an α = 0.05, and desired power of 0.90 (Cohen, 1988) suggested a total target sample size of 46 participants. Sixty-one adults participated; 13 identified as male (*M*_age_ = 26.77 years; SD_age_ = 5.94), 47 as female (*M*_age_ = 29.11 years; *SD* = 11.07), and one did not disclose their gender-sex (28 years). Participant recruitment, inclusion and exclusion criteria were identical to those in Study 1. There were 24 participants who reported having formally received an ASD diagnosis (7 males, *M*_age_ = 26.00, SD_age_ = 6.27; 16 females, *M*_age_ = 32.56, SD_age_ = 12.91; 1 non-disclosed, aged 28 years), and 37 otherwise NT participants (6 males, *M*_age_ = 27.67, SD_age_ = 4.84; 31 females, *M*_age_ = 27.32, SD_age_ = 9.74).

### Design, Materials, and Procedure

A 2 (participant group: *ASD, NT*) × 2 (emoji type: *happy, sad*) mixed-factors design was implemented via an online survey method to investigate participants’ emotional valence ratings of narrative sentences. Sentence-texts were identical to those of Robus et al. (2020). Emotional tone was assessed by an independent group of 62 participants. These individuals were presented the ‘naked’ written texts (without emoji) and filler materials, in a random order. Raters appraised them on a scale of 1 (highly negative)—5 (neutral)—9 (highly positive). The mean sentence valence ratings were considered within the appropriate parameters for neutrality (*M*_valence_ = 5.13, *SD*_valence_ = 0.54, min = 4.14, max = 5.94; for full details, see Robus et al., 2020). Thirty-six trials were presented, of which 18 included happy emoji and 18 sad. Emoji were randomly assigned to each neutral narrative sentence by first allocating each sentence-stimulus an identifier (1 to 36), then generating a pseudo-random sequence of 36 tokens—happy (0) or sad (1); the sentence identifier was then paired with its corresponding emoji token. To counterbalance the stimuli, a second list of stimuli was then created wherein the emoji type paired with each sentence was swapped (i.e., a Latin square design). That is, participants who viewed stimulus list 1 saw written stimulus *x* with emoji type *a*, whereas participants viewing stimulus list 2 saw written stimulus *x* with emoji type *b* (and vice versa). As in Study 1, to account for user familiarity, half of the emoji were presented in iOS format and half Android format. Example stimuli are presented in Table 3. Stimuli were presented in the same random order to each participant.

**Table 3:**
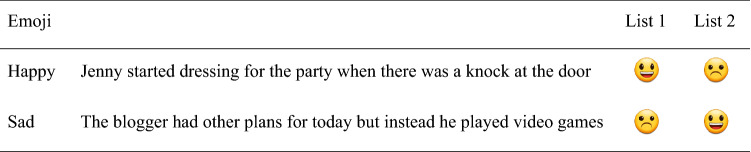
Study 2—example stimuli

Identical ethical protocols to those in Study 1 were followed. Text was presented in 14-point Times New Roman font on a white background; emoji were sized to align with the text, positioned one character-space to the right of sentences. Participants rated perceived valence using a seven-point Likert-type scale ranging from “*very negative”* (− 3) to “*very positive”* (+ 3). Each stimulus was displayed for an unlimited amount of time (i.e., until participants executed their response).

## Results

**Fig. 2:**
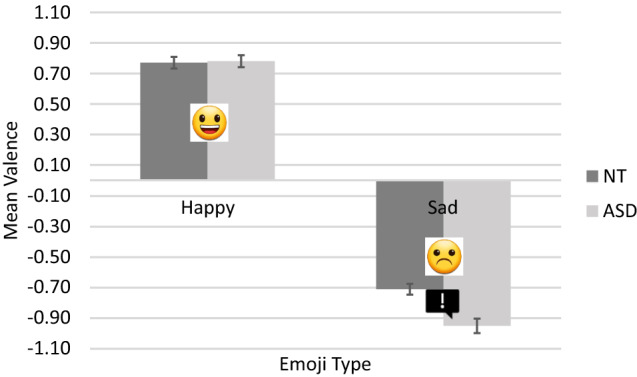
Mean valence ratings across emoji and groups. Error bars represent 5% error.  indicates a significant difference

Across participants and trials, there were 2,304 data points available for analysis. We used the ‘ordinal’ package in R to generate CLMMs (Christensen, 2019). Optimal random effect structures were identified using forward model selection (see Barr et al., 2013; Matuschek et al., 2017). Fixed effects were tested using likelihood-ratio tests comparing full and reduced models. Post-hoc tests were conducted using the ‘emmeans’ package (v1.4.8, 26/06/20; Lenth et al., 2020); significance thresholds were adjusted using the Bonferroni method. Descriptive statistics are presented in Table 4 and visualised in Fig. 2.

**Table 4:**
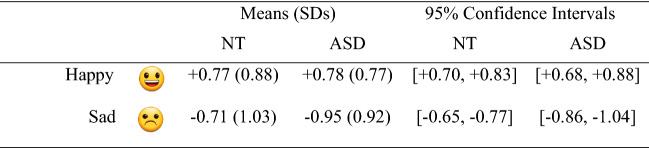
Means (SDs) and 95% confidence intervals of valence ratings across groups and emoji

Our model included random intercepts by participants [*χ*^2^ = 87.96, *df* = 1, *p* < 0.001] and by items [*χ*^2^ = 803.03, *df* = 1, *p* < 0.001]. Analysis revealed a non-significant effect of group on emotional valence ratings [*χ*^2^ = 3.33, *df* = 1, *p* = 0.068]. A significant effect of emoji type was found [*χ*^2^ = 47.51, *df* = 1, *p* < 0.001]; the mean rating for sentences + happy emoji was + 0.78, whereas the mean rating for sentences + sad emoji was -0.83.

A significant group × emoji type interaction was observed [*χ*^2^ = 8.30, *df* = 1, *p* = 0.004]. Follow-up comparisons revealed that NT and ASD-diagnosed participants rated sentences + happy emoji equally positively [*z* = -0.330, *p* = 0.741]; however, there was a significant between-group difference for sentences + sad emoji; ASD-diagnosed participants rated these stimuli more negatively (-0.95) than NT participants (-0.71) [*z* = 2.394, *p* = 0.017].

## Discussion

We proposed that emoji would bias perceived sentence sentiment toward the valence of the emoji; this was supported—for both emoji, confidence intervals indicated ‘non-neutrality’, albeit not powerfully. We observed—as predicted—differential effects of emoji on valence dependent on participant group; ASD-diagnosed participants rated otherwise neutral texts as more negative when presented with a sad emoji than NT participants. At a purely psycholinguistic level, these findings lend support to the theoretical perspective of compositionality—that the meaning of a message is determined by the meaning of the constituent parts of that message (e.g., Szabó, 2019).

Differences in the strength of emoji influence on valence ratings between Study 2 and previous research may be understood by comparing our third-person neutral narratives as with previous studies’ conversational dialogues (Willoughby & Liu, 2008). Following Social Information Processing theory (Walther, 1992), emoji are applied to develop and maintain online reciprocal relationships. Presenting third-person perspective sentences may influence readers’ ability to relate to subjects within the narrative, causing them to neglect the emoji and assess the sentence alone. However, research by Willoughby and Liu (2018) demonstrates attentional focus was routinely directed toward sentences including emoji, regardless of narrative sentence format. It is possible sentences were too neutral to strongly be impacted by the emoji sentiment, and that ASD-diagnosed and NT participants were performing at a ceiling. Our finding of enhanced emotionality ratings by ASD-diagnosed participants for text + negative emoji may reflect greater object personification of the emoji (White & Remington, 2019) however, it is unclear why this would be limited to the negative emoji condition.

Increased fixation durations on sentence-final emoji have been associated with semantic binding (Robus et al., 2020), implying a decision-making process, occurring when drawing conclusions on sentence sentiment. Given our sentences’ neutrality, readers, by attempting to incorporate emoji into semantic binding, may have experienced incongruence between emoji and text leading to an ultimate decision to discard emoji, concluding they did not add to sentence valence. Walther and D’Addario (2001) demonstrated emotionality can be increased when text sentiment and emoji suggest similar valence (negative-negative). Where text/emoji appear incongruent, emoji may be mistaken as sarcasm or irony markers (Thompson & Filik, 2016).

### General Discussion

Study 1 demonstrated that ASD-diagnosed and NT controls showed differential performance when classifying emoji expressions of fear, surprise, and sadness. Identification of happy, disgusted, and angry emoji were equivalent across groups. In Study 2, we showed that perceived emotional sentiment of otherwise neutral third-person narrative texts was influence by emoji, and that although ASD-diagnosed and NT participants were similarly influenced by happy/positive emoji, ASD-diagnosed participants rated sentences + sad emoji more-negatively than NT controls.

### Theoretical and Practical Implications

Processing differences for particular emotions have both theoretical and practical implications. Study 1 demonstrates that ASD-diagnosed individuals were less-proficient in recognising emoji depicting negative affect. Further research is needed to understand the relationship between how individuals use emoji in their own lives and how these map against ‘typical’ or intended uses of these emoji. One interpretation of these findings might be that emoji are more-ambiguous and/or are used ‘differently’ by autistic persons, and this ties in with Milton’s (2012) double empathy perspective; it’s not that these participants ‘couldn’t’ recognise these emoji, rather that ‘what’ they use these emoji for is different to what the ‘classic’ interpretation/Ekman-type labelling would suggest. Our ‘confusion matrix’ (see Supplementary Material B) would suggest that such divergence in perception is most-obvious among ASD-diagnosed individuals for the emoji we had chosen to represent disgust and surprise. In terms of exploring how well models of autism that posit a ‘single-minded’ attentional system (e.g., Murray et al., 2005) explain the differential effects observed in the current Study 2, future research would be required that makes use of, for example, eye tracking technology (as in Robus et al., 2020). Such research could compare the allocation of visual attention during the processing of sentences + emoji by autistic and non-autistic persons, delineating the relative processing of written and emoji components of the stimuli.

As with any study, we must consider the possibility of Type I/Type II errors. We defended against these in many ways (e.g., Forstmeier et al., 2016). We determined a priori hypotheses. We performed a priori power calculations and sampled accordingly. We adjusted our analyses to account for multiple comparisons, particularly in Study 1. We performed state-of-the-art analyses (Taylor et al., 2021), rather than falling back on non-optimal analyses such as those based around general linear models or non-parametric tests. It must be recognised, however, that in many cases our effect sizes are not particularly large, either in statistical or ‘real’ terms. For example, it is possible that incorrectly executed responses by neurodivergent participants account for the significance and size of the effect seen in Study 1 ‘fearful’ data. However, the pattern of confusion data (see Supplementary Material B) suggests that there is too much systematicity to this data, rather than random participant response execution error (this is additionally unlikely due to the presentation of trial items/response options as-randomised). Similarly, it could be argued that the non-difference between participant groups in Study 1 ‘angry’ data (*p* = 0.053) is problematic and potentially influenced by participant execution errors and/or ‘lucky guesses’. However, this seems unlikely given the robustness of the analysis (e.g., Taylor et al., 2021). Study 2 ‘message rating’ data could also have been influenced by participant execution error; however, this also seems very unlikely. All trials in both studies were self-paced, so the risk of a speed-accuracy trade-off is virtually nil, and the risk of ‘performance anxiety’ or ‘observation anxiety’ is also effectively nil as data was collected online and remotely.

Computer-mediated technologies have changed the way individuals communicate, becoming paralinguistic languages with their own grammar and diction. The boundaries of visual and verbal communications, are at best, blurred within online environments. Social Information Processing theory (Walther, 1992) states that such cues within online communications are intended to develop and maintain social relationships (Rodrígues et al., 2017). Skovholt et al. (2014) highlight how emoji function as context cues, attitude markers, and social relationship organisers (e.g., decreasing formality or ambiguity). Paradoxically, internet culture is governed by layers of irony and subtle nuance, so much so that one piece of content may derive multiple different meanings, depending on individuals’ assimilation-level within a community, or context in which content is observed. Often, emoji and the meanings attributed to them, morph into something almost unrecognisable and dissociated from their intended derivatives and original contexts (e.g., the aubergine).

As Milton (2012) has compellingly argued, challenges surrounding communication and understanding between autistic and non-autistic people are not one-sided—suggesting a double empathy problem, resultant from the different perspectives of the two communicators. In order to more fully understand this double empathy problem, further research of the type described in this paper is needed—examining how communicators (neurodivergent or NT) use emoji when communicating, how these emoji are perceived, and how emoji and text interact to shape perceptions of messages and senders. Furthermore, this needs to take place in real time with measures taken from both senders and recipients as to their true and perceived feelings/intentions/states. This research would also need to consider the relationship between communicators—that is are both NT, are both neurodivergent, or is one communicator NT and the other ASD-diagnosed? This ties in with research by, for example, Gernsbacher et al. (2017) and Crompton et al. (2019) which demonstrated the importance of ‘status’ (in-group/out-group) dynamics on interpersonal perceptions and rapport.

### Limitations and Suggested Future Research Directions

Our method for determining participant group affiliation was blunt and required participants to identify based on a formal diagnosis by a professional/team of professionals. Due to practical and ethical constraints, we were unable to verify these diagnoses and rely upon participant honesty. Furthermore, this approach is somewhat hindered by the potential inconsistency in the approaches used to reach diagnosis; for example, inconsistent qualification standards/training/experiences and possible biases of the individuals and teams involved. This is not just a problem for our research, of course, but a problem in the real world too. At the design stage, we considered incorporating a measure of stereotypical ASD traits, such as the AQ-10 (Allison et al., 2012). However, the psychometric properties of this measure have been questioned recently (e.g., Bertrams, 2021; Taylor et al., 2020). Furthermore, the items used in measures such as these are not always relevant to individuals diagnosed with ASD, and/or are certainly not uncommon in so-called typical samples.

Our participants were not matched on characteristics such as age or IQ. Previous research by Uljarevic and Hamilton (2012) suggests that recognition differences were not age or IQ sub-group-specific, and their meta-analysis did not suggest that increased age would equate to increased performance. Furthermore, IQ measures are only reliable in acknowledging that ASD-diagnosed participants perform at their expected mental age, but do not provide a means of directly comparing chronologically same-aged individuals. Matching age and IQ may not necessarily enhance a study, as the ASD-diagnosed population in general does not perform equally in testing to their NT peers (due to the testing context itself).

We did not include measures of other individual difference dimensions that may have been illuminating. These include co-morbid anxiety and alexithymia. Co-morbid anxiety is present in approximately 40% of individuals diagnosed with ASD (Zaboski & Storch, 2018). There is an inconsistent pattern of findings in relation to the relationship between anxiety and the processing of emotion; certain studies suggest a detrimental effect of anxiety on facial expression recognition tasks (e.g., Cooke et al., 2016; Li, 2013), whereas others indicate that anxiety can facilitate understanding of negative facial expressions (e.g., Cooper et al., 2009) due to hypervigilance. Future research should incorporate more elegant, multi-faceted measures of individual differences, for both neurodivergent and NT samples. Furthermore, we did not ascertain differences in social variance or communicative ability across our participants; however, our sophisticated modelling, which allows for simultaneous modelling of fixed and random effects (such as the random effect of ‘participant’ would have accounted for this, to an extent. Future research in this area could perhaps attempt to quantify this and incorporate it as a fixed factor into the analysis.

Emoji are often ambiguous and standardised emoji norms, representative of different populations, are somewhat lacking. The current study made use of written texts that were pre-tested to ensure emotional neutrality. Recent work (Boutet et al., 2021) has suggested the (in)congruence of emoji and accompanying text-sentiment influences message and person perceptions, however, their analytical method (ANOVA) was sub-optimal given the nature of the ratings data in their study. It is unclear whether neuro-divergent/neuro-typical individuals would demonstrate such (in)congruence effects equally, and further research is required to explore this. Stakeholders who utilise emoji in communication (e.g., educators, and/or those working with non-verbal individuals) should recognise the relationship between emoji and text on such perceptions. Future research is encouraged to examine emoji effects on specific target-word perceptions in neurodiverse groups, and if these differentiate from NT participants (e.g., contextual fit of specific words, predictability/plausibility judgements, arousal ratings). Although our studies included two different emoji formats (iOS, Android), this variable was not analysed. Future researchers may examine whether differences exist in emoji processing across platforms. This may identify graphical representations associated with greatest interpretation-consistency and improve implementation validity in therapeutic and educational environments.

### Gender-Sex

Considering participant gender-sex, Loomes et al. (2017) reported a ratio of approximately three males to every female diagnosed with ASD. Watkins et al.’s (2014) meta-analysis examined 607 studies involving ASD-diagnosed participants, finding that 86% of participants from 2010 to 2012 were male. Our studies are atypical, in that the majority of participants identified as female (in both ASD-diagnosed and NT groups). Previous research suggests ASD-diagnosed females exhibit similar emotional processing skills to NT males (Baron-Cohen et al., 2011; Kok et al., 2016), therefore our imbalanced groups should not be problematic. A rudimentary re-analysis of our data shows no suggestion of gender-sex effects (nor group × gender-sex interactions) on emoji identification ability or valence ratings.

### Conclusions

In summary, our studies generally support processing differences between ASD-diagnosed and NT individuals. It is clear from our results that both ASD and NT individuals are well-above chance levels when identifying which emoji represent which of Ekman et al.’s (1972) classic universal emotions; however, inter-group differences in the consistency or concreteness with which these as categorised suggest that there may be greater ambiguity/divergence among ASD-diagnosed individuals’ perceptions. There is much to be learned about how we use and interpret these increasingly prevalent digital communication devices. Our results suggest that emoji modify recipients’ perceptions of emotional tone within written texts, for both NT and ASD-diagnosed individuals, again with inter-group differences related to negative/sad emoji. However, we must qualify our conclusions, given that our findings were obtained using a reasonably small set of emoji, and a reasonably small set of third-person neutral narrative sentences (as opposed to, for example, social interaction communications such as instant messaging and/or email). Communicators must be aware that the use of emoji can modify the perceived tone of their messages which can in turn modify the experience of the user, and potentially impact upon their own emotional experience and subsequent behaviours.

## Supplementary Information

Below is the link to the electronic supplementary material.

Supplementary file1 (DOCX 13 kb)

Supplementary file2 (DOCX 15 kb)
