# Supplementary material for: Emoji Identification and Emoji Effects on Sentence Emotionality in ASD-Diagnosed Adults and Neurotypical Controls
Source: J Autism Dev Disord. 2022 Apr 12;53(6):2514–28. doi: 10.1007/s10803-022-05557-4 (PMC10229741; doi:10.1007/s10803-022-05557-4)
Supplement: Supplementary file 1 — Supplementary file1 (DOCX 13 kb) [file 10803_2022_5557_MOESM1_ESM.docx]

**Supplementary Material A**

**Table A1.** Comparisons of accuracy across emoji and groups

|  | | **NT participants** | | **ASD participants** | |
| --- | --- | --- | --- | --- | --- |
| **Emoji** | | ***z-ratio*** | ***p*** | ***z-ratio*** | ***p*** |
| Happy | Disgusted | 6.330 | **<.0001** | 4.432 | **.0001** |
|  | Fearful | 3.203 | **.0171** | 3.657 | **.0035** |
|  | Sad | 1.483 | .6752 | 2.063 | .3068 |
|  | Surprised | 1.481 | .6762 | 1.902 | .4007 |
|  | Angry | 0.694 | .9826 | 1.721 | .5175 |
| Disgusted | Fearful | 4.444 | **.0001** | 1.862 | .4256 |
|  | Sad | 5.012 | **<.0001** | 4.602 | **.0001** |
|  | Surprised | 5.011 | **<.0001** | 4.757 | **<.0001** |
|  | Angry | 6.141 | **<.0001** | 4.898 | **<.0001** |
| Fearful | Sad | 3.315 | **.0118** | 3.069 | **.0262** |
|  | Surprised | 3.313 | **.0119** | 3.272 | **.0136** |
|  | Angry | 3.504 | **.0061** | 3.471 | **.0069** |
| Sad | Surprised | 0.001 | >.9999 | 0.266 | .9998 |
|  | Angry | 0.953 | .9324 | 0.548 | .9941 |
| Surprised | Angry | 0.952 | .9328 | 0.284 | .9998 |
