# Supplementary material for: Emoji Identification and Emoji Effects on Sentence Emotionality in ASD-Diagnosed Adults and Neurotypical Controls
Source: J Autism Dev Disord. 2022 Apr 12;53(6):2514–28. doi: 10.1007/s10803-022-05557-4 (PMC10229741; doi:10.1007/s10803-022-05557-4)
Supplement: Supplementary file 2 — Supplementary file2 (DOCX 15 kb) [file 10803_2022_5557_MOESM2_ESM.docx]

**Supplementary Material B**

**Table B1.** Confusion matrix of responses (%) by participant group and emoji type

| **NT** | **Happy** | **Disgusted** | **Fearful** | **Sad** | **Surprised** | **Angry** |
| --- | --- | --- | --- | --- | --- | --- |
| Happy | 95.61 | 0.00 | 0.00 | 0.00 | 0.00 | 0.00 |
| Disgusted | 0.00 | 46.49 | 3.51 | 0.00 | 0.00 | 0.88 |
| Fearful | 0.00 | 15.79 | 79.82 | 0.88 | 0.88 | 0.88 |
| Sad | 0.00 | 16.67 | 5.26 | 99.12 | 0.00 | 0.88 |
| Surprised | 3.51 | 0.00 | 10.53 | 0.00 | 99.12 | 0.00 |
| Angry | 0.00 | 21.05 | 0.88 | 0.00 | 0.00 | 97.37 |
| **ASD** | **Happy** | **Disgusted** | **Fearful** | **Sad** | **Surprised** | **Angry** |
| Happy | 98.39 | 0.00 | 0.00 | 0.00 | 0.00 | 0.00 |
| Disgusted | 0.00 | 43.55 | 3.23 | 4.84 | 4.84 | 6.45 |
| Fearful | 0.00 | 24.19 | 61.29 | 8.06 | 6.45 | 1.61 |
| Sad | 0.00 | 11.29 | 4.84 | 87.10 | 0.00 | 1.61 |
| Surprised | 1.61 | 3.23 | 30.65 | 0.00 | 88.71 | 0.00 |
| Angry | 0.00 | 17.74 | 0.00 | 0.00 | 0.00 | 90.32 |

Note. Percentages rounded to 2DP. NT %age out of a possible total of 114 choices (57 participants identifying two of each emoji); ASD %age out of a possible 62 choices (31 participants identifying two of each emoji).
